# Clinical significance of anti-Epstein–Barr virus antibodies in systemic chronic active Epstein–Barr virus disease

**DOI:** 10.3389/fmicb.2023.1320292

**Published:** 2024-01-08

**Authors:** Miwako Nishio, Minori Saito, Mayumi Yoshimori, Yuki Kumaki, Ayaka Ohashi, Eri Susaki, Ichiro Yonese, Megumi Sawada, Ayako Arai

**Affiliations:** ^1^Department of Hematology and Biophysical Systems Analysis, Graduate School of Medical and Dental Sciences, Tokyo Medical and Dental University (TMDU), Tokyo, Japan; ^2^Department of Frontier Medicine, Institute of Medical Science, St. Marianna University School of Medicine, Kanagawa, Japan; ^3^Department of Hematology, Tokyo Medical and Dental University (TMDU), Tokyo, Japan; ^4^Department of Nutrition, Tokyo Kasei University, Tokyo, Japan; ^5^Department of Hematology and Oncology, St. Marianna University School of Medicine, Kanagawa, Japan

**Keywords:** systemic chronic active Epstein–Barr virus disease, sCAEBV, anti-Epstein–Barr virus antibody, VCA-IgG, EBNA

## Abstract

Systemic chronic active Epstein–Barr virus disease (sCAEBV) is a rare and fatal neoplasm, involving clonally proliferating Epstein–Barr virus (EBV)-infected T cells or natural killer cells. Patients with sCAEBV have abnormal titers of anti-EBV antibodies in their peripheral blood, but their significance is unknown. We retrospectively investigated titers and their relationship with the clinical features of sCAEBV using the data collected by the Japanese nationwide survey. Eighty-four patients with sCAEBV were analyzed. The anti-EBV nuclear antigen (EBNA) antibody, targeting EBNA-expressing EBV-positive cells, was found in 87.5% of children (<15 years old), 73.7% of adolescents and young adults (15–39 years old), and 100% of adults (≥40 years old). Anti-EBNA antibody titers were significantly lower and anti-VCA-IgG antibody titers significantly higher in patients with sCAEBV than those in healthy controls (*p* < 0.0001). Patients with high anti-VCA-IgG and anti-early antigen-IgG antibody (antibodies against the viral particles) levels had significantly better 3-year overall survival rates than those with low titers, suggesting that patients with sCAEBV have a reduced immune response to EBV-infected cells.

## Introduction

Systemic chronic active Epstein–Barr virus disease (sCAEBV) is an Epstein–Barr virus (EBV)-infected T-or natural killer (NK) cell neoplasm, according to the World Health Organization (WHO) classification of tumors of hematopoietic and lymphoid tissues in 2022 (Alaggio et al., 2022). Why EBV persistently infects T or NK cells, leading to their immortalization and clonal expansion in sCAEBV, is unknown. Moreover, no effective drug has been developed to eradicate EBV-infected T or NK cells in patients with sCAEBV (Yonese et al., 2020). Thus, there is an urgent need to elucidate the pathological mechanism and develop therapeutic agents.

In sCAEBV, dysfunction of EBV-specific cytotoxic T cells occurs (Fujieda et al., 1993; Shibayama et al., 2017). In addition, congenital immunodeficiency with *FAS, IL2RG*, or *PRF1* gene mutations can be complicated by sCAEBV-like EBV-positive T- or NK-cell lymphoproliferative disorders (Katano et al., 2004; Sekinaka et al., 2017; Ishimura et al., 2019; Tanita et al., 2019). These results indicate that immunodeficiency against EBV or EBV-infected cells underlies the pathogenesis of sCAEBV. However, patients with sCAEBV present with hypergammaglobulinemia and elevated anti-EBV antibody levels, particularly those against viral capsid antigen (VCA), a viral protein, thereby suggesting a hyperimmune state of the disease (Okano et al., 2005).

EBV is a ubiquitous virus and belongs to the human herpes virus family. EBV mainly targets B lymphocytes, but also infects T cells and NK cells. The genome of EBV encodes a variety of genes, and the humoral response produces antibodies to the products of these genes. Anti-VCA antibodies involve three immunoglobulin classes, IgG, IgA and IgM. All anti-VCA antibodies appear in the acute phase of EBV infection, and then only the anti-VCA-IgG antibody remains positive after the acute phase. EBNA proteins are synthesized in the latent phase. Anti-EBNA antibodies appear 3–6 weeks after EBV infection and remain present throughout life. Thus, the detection of anti-EBNA antibody indicates past EBV infection or recovery. Early antigen-diffuse and restrict complex (EA-DR) is synthesized in the lytic phase of the EBV replication. EA-DR is composed of two components, EA-D and EA-R. Usually, Anti-EA antibodies appear in the acute phase and then decline to undetectable levels. The high titers of anti-EA antibody can be detected in the different diseases and in healthy individuals (Smatti et al., 2018). Not all patients with sCAEBV present with unusual patterns of anti-EBV antibodies (Xiao et al., 2016). The association between EBV-associated antibodies and sCAEBV is unclear, and there is no common pattern in all cases (Kimura et al., 2001; Okano et al., 2005; Kimura, 2006).

To evaluate the significance of EBV antibody titers in sCAEBV, we investigated the association between EBV antibody titers in serum samples and clinical features, including prognosis, in patients with sCAEBV according to the WHO 2022 classification.

## Materials and methods

### Study design

This is an observational study of clinical information. We conducted a retrospective study based on a nationwide survey conducted from 2016 to 2018 by the Japanese Study Group of sCAEBV, supported by the Japanese Agency for Medical Research and Development, to identify the clinical features and treatments for sCAEBV. The details of patient collection have been previously described (Yonese et al., 2020). For the analysis, we selected patients whose anti-EBV antibody titers measured using the fluorescent-labeled antibody (FA) methods in standardized laboratories were available. Since the quantification of the titers of anti-EBV antibodies is covered by health insurance and widely performed as a general examination, the diagnostic agents are verified annually by The Japanese Society of Clinical Virology. Specifically, each antibody was reacted in serum with cultured cells and fixed on a glass slide, expressing each antigen of human origin. After washing off excess serum, anti-human/rabbit polyclonal antibodies labeled with fluorescent dyes were added. Each antibody was detected by observation under a fluorescence microscope and the antibody titer was quantified at the highest dilution of the detected sample. Antibody titers of patients were measured by SRL, Inc., BML, Inc., and other Japanese clinical laboratories. Plasma of healthy individuals was measured by BML.

Patients with anti-VCA-IgG antibodies were considered to be EBV seropositive. This is because anti-VCA-IgG antibodies are known to gradually rise during the acute phase of EBV infection and persist throughout life, whereas anti-EBNA antibodies are persistently detected during recovery from the initial EBV infection. If either one is positive, the patient is considered to be already infected with EBV (Schillinger et al., 1993). However, anti-EBNA antibody was reported to be negative in 5–10% of healthy individuals who were positive for the anti-VCA-IgG antibody (Smatti et al., 2018). Thus, the anti-VCA-IgG antibody more accurately indicates past infection with EBV.

### Diagnostic criteria

The patients were diagnosed with sCAEBV based on the following diagnostic criteria, in line with the WHO 2022 classification (Kimura et al., 2012; Yonese et al., 2020; Alaggio et al., 2022):

1. Elevated EBV DNA load in peripheral blood (PB) > 10^2.5^ copies/μg DNA,
2. EBV infection of T or NK cells in the PB or affected tissues,
3. Systemic inflammatory symptoms, such as infectious mononucleosis-like conditions persisting for >3 months, and.
4. Exclusion of other possible diagnoses known immunodeficiency, malignancy, or autoimmune disorders.

### Healthy controls

In this study, we included 220 healthy student volunteers from the adolescent and young adult (AYA) generation. Students with any disease were excluded. We collected their blood samples from 2018 to 2020. Among them, 178 volunteers (103 males and 75 females) who were positive for the anti-VCA-IgG antibody, were used as EBV-positive healthy controls. We measured anti-EBV antibodies, the anti-VCA-IgG antibody and anti-EBV nuclear antigen (EBNA) antibody, using the FA methods in standardized laboratories. All fresh samples were measured on the collection day.

### Statistical analysis

The Mann–Whitney U test was used to compare the distributions of anti-VCA-IgG and anti-EBNA antibodies between healthy individuals and patients. In the group of patients, data of three age groups [children (< 15 years), AYA (15–39 years), and adults (> 39 years)] were compared. The log-rank test was used for survival analysis. Statistical significance was set at *p* < 0.05. All statistical analyzes were performed using GraphPad Prism version 8 (GraphPad Software Inc., Boston, MA, United States).

### Ethics statement

The study protocol of the nationwide survey of the sCAEBV was approved by the ethics committees of Tokyo Medical and Dental University and St. Marianna University School of Medicine.

The analysis of the samples from healthy donors was approved by the ethics committees of Tokyo Medical and Dental University, St. Marianna University School of Medicine, and Tokyo Kasei University and registered at University hospital Medical Information Network (UMIN) Center (#UMIN000032099). The donors provided written informed consent to participate in this study.

### Data sharing statement

The data that support the findings of this study are available upon request from the corresponding author, AA. The details of the nationwide survey have been published previously (Yonese et al., 2020).

## Results

### Clinical features of sCAEBV patients

Among the 100 patients analyzed in the nationwide survey of patients with sCAEBV, 84 patients whose anti-EBV antibodies were examined using the FA method were included in this study. A flowchart of patient selection is shown in Supplementary Figure S1. The clinical findings of the patients are summarized in Table 1. The study included 43 males and 41 females, aged 3–78 years (median: 23.0 years). The patient samples were collected at multiple facilities, and the methods of detecting EBV-infected lymphocyte phenotypes were as follows: antibody-conjugated magnetic bead sorting (*n* = 55), flow cytometry (*n* = 14), and histopathology using *in situ* hybridization for EBV-encoded small RNA (*n* = 8). Detailed methods for four patients were not available. The EBV-infected cell phenotypes were as follows: CD4-positive cells (23, 27.4%), CD8-positive cells (11, 13.1%), CD56-positive cells (25, 29.8%), and γδT-cells (2, 2.4%). EBV was detected in multiple T and NK cells from 18 patients. Hypersensitivity to mosquito bites and hydroa vacciniforme-like skin eruptions were observed in 20 (23.8%) and 5 (6.0%) patients, respectively. Moreover, 21 patients (25.0%) had accompanied hemophagocytic lymphohistiocytosis, a fatal complication of sCAEBV. No significant differences in clinical features were observed between the patients selected for the present study and 100 patients of the nationwide survey, the population group. A total of 220 donors, were considered as healthy controls, of which 178 had anti-VCA-IgG antibodies and were considered as EBV seropositive.

**Table 1:** Comparison of patient characteristics and clinical findings between this study and a previous report (Yonese et al., 2020).

	Patients (*N* = 84)	The population (*N* = 100) *	p**
Male/female sex, *n*	43/41	53/47	0.8824
Age at diagnosis, range (median), y	3–78 (23)	1–78 (21)	
Child (aged <15 y)	26 (17/9)	37 (25/12)	0.4370
AYA (15 ~ 39 y)	42 (23/19)	46 (24/22)	0.6573
Adult (aged >39 y)	16 (3/13)	17 (4/13)	0.8473
EBV-infected cell type			
CD4	23 (27.4%)	25 (25.0%)	0.7385
CD8	11 (13.1%)	13 (13.0%)	>0.9999
CD56	25 (29.8%)	28 (28.0%)	0.8705
γδT	2 (2.4%)	3 (3.0%)	>0.9999
CD56- NK	0 (0.0%)	2 (2.0%)	0.5011
CD4 + CD8	6 (7.1%)	6 (6.0%)	0.7733
CD4 + CD56	3 (3.6%)	4 (4.0%)	>0.9999
CD8 + CD56	1 (1.2%)	1 (1.0%)	>0.9999
CD4 + CD8 + CD56	4 (4.8%)	6 (6.0%)	0.7568
Others	4 (4.8%)	7 (7.0%)	0.8203
NA	5 (6.0%) ***	5 (5.0%) ***	
Symptoms and signs at diagnosis			
Fever	70 (83.3%)	85 (85.0%)	0.8400
Hepatosplenomegaly	59 (70.2%)	64 (70.3%)	>0.9999
Lymphadenopathy	44 (52.4%)	48 (52.7%)	>0.9999
Cardiac dysfunction	7 (8.3%)	8 (8.8%)	>0.9999
Aneurysm	7 (8.3%)	8 (8.8%)	>0.9999
Gastrointestinal symptom	7 (8.3%)	7 (7.7%)	>0.9999
Neurological symptoms	7 (8.3%)	7 (7.7%)	>0.9999
Vasculitis	6 (7.1%)	6 (6.6%)	>0.9999
Uveitis	4 (4.8%)	4 (4.4%)	>0.9999
HLH	21 (25.0%)	24 (26.4%)	0.8641
HMB	20 (23.8%)	25 (25.0%)	0.8653
HV-like eruption	5 (6.0%)	9 (9.0%)	0.5794
Neutropenia (< 1,000 /μL)	11 (13.9%)	14 (15.6%)	0.6708
Anemia (< 9 g/dL)	12 (14.6%)	12 (13.6%)	>0.9999
Thrombocytopenia (< 10 × 10^4^/μL)	23 (27.7%)	27 (30.3%)	0.7391
High ALT (> 128 U/L)	26 (31.7%)	27 (30.7%)	>0.9999
High sIL-2R (> 2,400 U/mL)	16 (25.8%)	17 (26.6%)	>0.9999
Chemotherapy	20	20	0.8619
Chemotherapy + HSCT	43	47	
HSCT	12	12	

NA, the detailed phenotype of the infected cells was not available; HLH, hemophagocytic lymphohistiocytosis; HMB, Hypersensitivity to mosquito bites; HV, hydroa vacciniforme; HSCT, hematopoietic stem cell transplantation; ALT, alanine transaminase; AYA, adolescent and young adult. *Patients analyzed in the nationwide survey (Yonese et al., 2020). **Fisher’s exact test. ***1 and 4 patients were described to be CD3- and NK-infected, respectively; derailed phenotypes were not available.

### EBV-associated antibodies in sCAEBV patients

**Figure 1:**
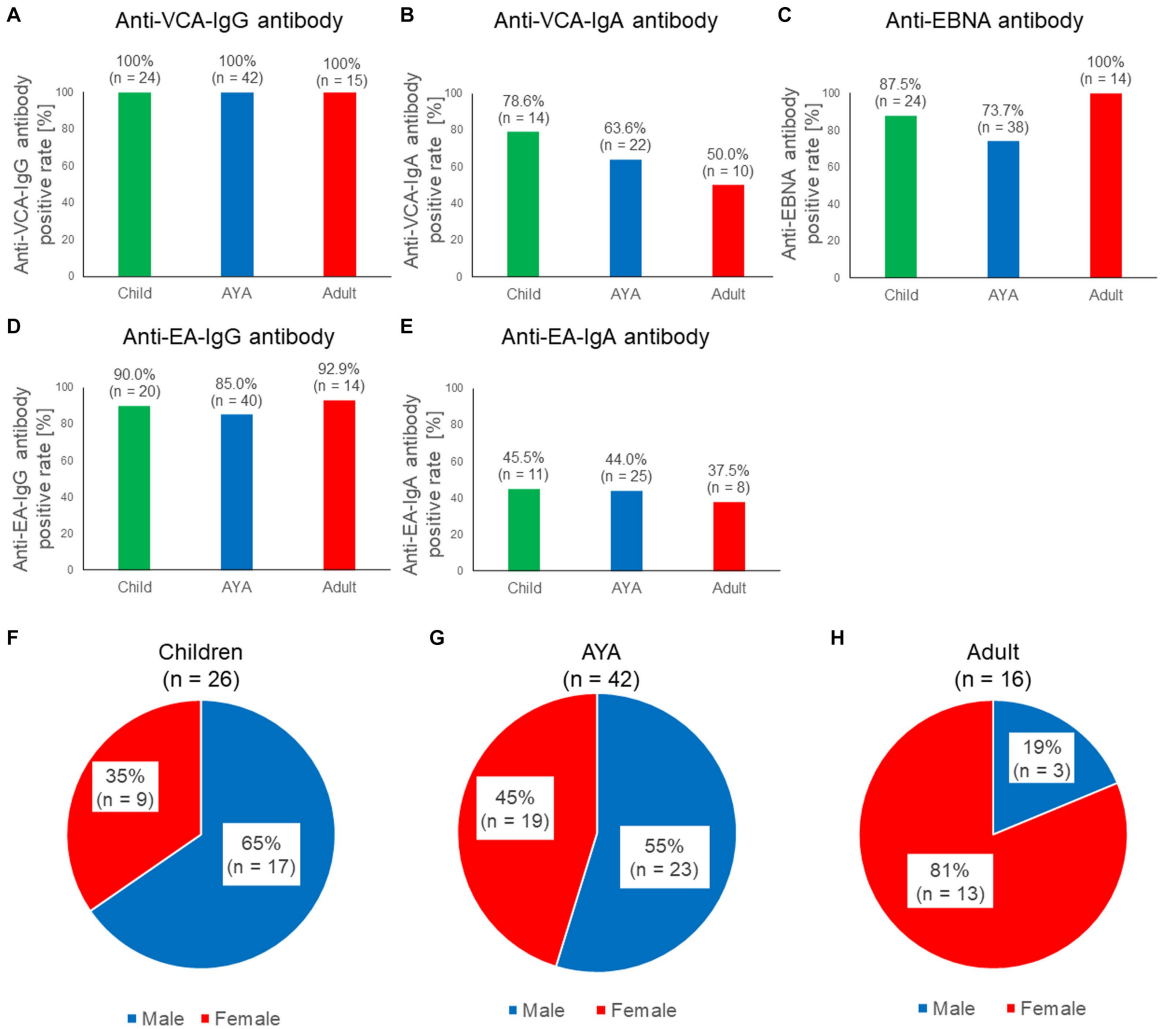
Characteristics of the five anti-EBV antibodies in patients with sCAEBV. **(A–E)** Comparisons of the seropositive rate among three generations for five anti-EBV antibodies: anti-VCA-IgG antibody, anti-VCA-IgA antibody, anti-EBNA antibody, anti-EA-IgG, and anti-EA-IgA. Green indicates children, blue indicates AYA, red indicates adults. The number of available samples is shown at the top of the graphs. **(F–H)**. Ratios of males and females with sCAEBV among children, AYA, and adults. Blue indicates males, red indicates females. AYA, adolescent and young adult; EA, anti-early antigen; EBNA, EBV nuclear antigen; EBV, Epstein–Barr virus; sCAEBV, systemic chronic active Epstein–Barr virus disease; VCA, viral capsid antigen.

We investigated the positive rates of five EBV-associated antibodies in patients with sCAEBV to clarify the characteristics of each antibody in this disease. As our previous study revealed that the prognosis and sex differed based on the age of patients with sCAEBV, we examined the antibody titers in different age groups (Yonese et al., 2020). The number of patients in each age group is presented in Table 1. The positive rate of anti-EBV antibodies based on age is shown in Figures 1A–E. All patients tested positive for anti-VCA-IgG antibodies (Figure 1A); however, the positive rates of the anti-VCA-IgA antibody differed based on age as follows: 78.6% in children, 63.6% in AYA, and 50.0% in adults (Figure 1B). Although the difference was not statistically significant, the positive rates of the anti-VCA-IgA antibody tended to decrease with age. In contrast, 87.5, 73.7, and 100% of the children, AYA, and adults (Figure 1C), respectively, tested positive for the anti-EBNA antibody; thus, the positive rate in adults tended to increase by age. As shown in Figures 1D–E, no significant differences were observed in the positive rate of the anti-early antigen (EA) antibodies based on age. The sex distribution of the patients by age are shown in Figures 1F–H. Briefly, 65, 55, and 19% of children, AYA, and adults respectively, were males, indicating that childhood and adult sCAEBV cases have different characteristics.

As shown in Figure 1C, no anti-EBNA antibody-negative patients were over 40 years of age. The positive rates of the anti-EBNA antibodies by generation and sex are shown in Supplementary Figure S2A. In males, 82.4, 80.0, and 100% of children, AYA, and adults, respectively, tested positive for the anti-EBNA antibody. In females, 100, 66.7, and 100% of children, AYA, and adults, respectively, tested positive for the anti-EBNA antibody. Anti-EBNA antibody titers classified by generation and sex are shown in Supplementary Figure S2B. No significant differences were observed between the groups (*p* = 0.3885). Anti-VCA-IgG and anti EA-IgG antibody titers classified by sex are shown in Supplementary Figures S2C,D. No significant differences were observed between the groups (*p* = 0.9183, 0.3617). In addition, there were no significant differences in antibody titers with age (Supplementary Figure S3). As shown in previous reports (Kimura et al., 2001), this study also confirmed that anti-VCA-IgG and anti-EA-IgG antibody titers were significantly higher in the T-cell type of sCAEBV than in the NK-cell type of sCAEBV (Supplementary Figure S4).

### Comparison of EBV antibody titers between age-matched patients with sCAEBV and healthy controls

**Figure 2:**
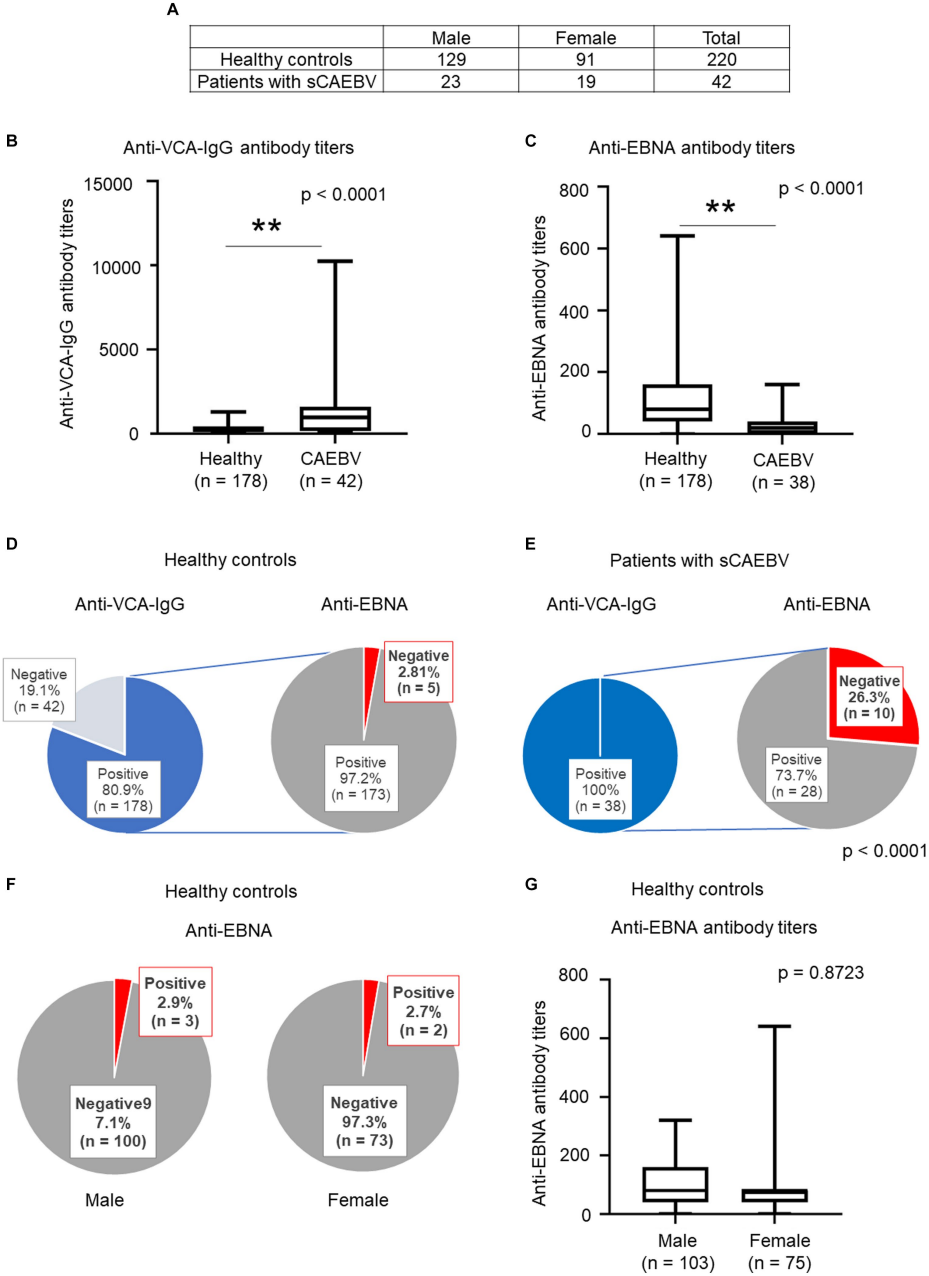
Comparison of anti-EBV antibodies among healthy young adults and age-matched AYA patients with sCAEBV **(A)** Sex of healthy controlsand patients with sCAEBV. **(B,C)** Comparison of the distribution of anti-VCA-IgG and anti-EBNA antibody titers carried out using the Mann–Whitney U test** *p* < 0.001 compared with the control. **(D,E)** Comparison of seroprevalence of anti-VCA-IgG and anti-EBNA antibody. The left graphs show the seroprevalence of anti-VCA-IgG. Bule indicates positive rate, while light gray indicates negative rate. The graphs on the right show the negative rates of the anti-EBNA antibody among individuals who were positive for the anti-VCA-IgG antibody. Red indicates the negative rate for the anti-EBNA antibody among individuals with the anti-VCA-IgG antibody. Dark gray indicates the positive rate for the anti-EBNA antibody or for both the anti-VCA-IgG and anti-EBNA antibody. **(F,G)** Comparison of anti-EBNA antibody positive rates and titers based on sex in healthy controls. EBNA, EBV nuclear antigen; EBV, Epstein–Barr virus; sCAEBV, systemic chronic active Epstein–Barr virus disease; VCA, viral capsid antigen.

Next, we compared the titers of the anti-VCA-IgG and anti-EBNA antibody between the healthy controls and age-matched patients with AYA sCAEBV (15–39 years old). Healthy controls consisted of 129 males and 91 females (Figure 2A). As shown in Figure 2B, the anti-VCA-IgG antibody titers were significantly higher in patients with sCAEBV than that in healthy controls (*p* < 0.0001; Figure 2B). In contrast, the anti-EBNA antibody titer of the healthy controls was higher than that of the patients (Figure 2B, *P* < 0.0001). A significant difference was observed in the negative rate of anti-EBNA antibodies between healthy controls and patients (*p* < 0.0001): 2.8% of the healthy controls tested negative for the anti-EBNA antibody (Figure 2D), while 26.3% of the patients with sCAEBV tested negative for this antibody (Figure 2E). To examine sex preference with respect to the positive rate of anti-EBNA antibodies, we analyzed the rate by sex in the healthy control group. No significant difference was observed in the rates and titers of anti-EBNA antibodies between males and females in the control group (Figures 2F,G).

### Relation between prognosis and anti-EBV antibody titers of patients with sCAEBV

**Figure 3:**
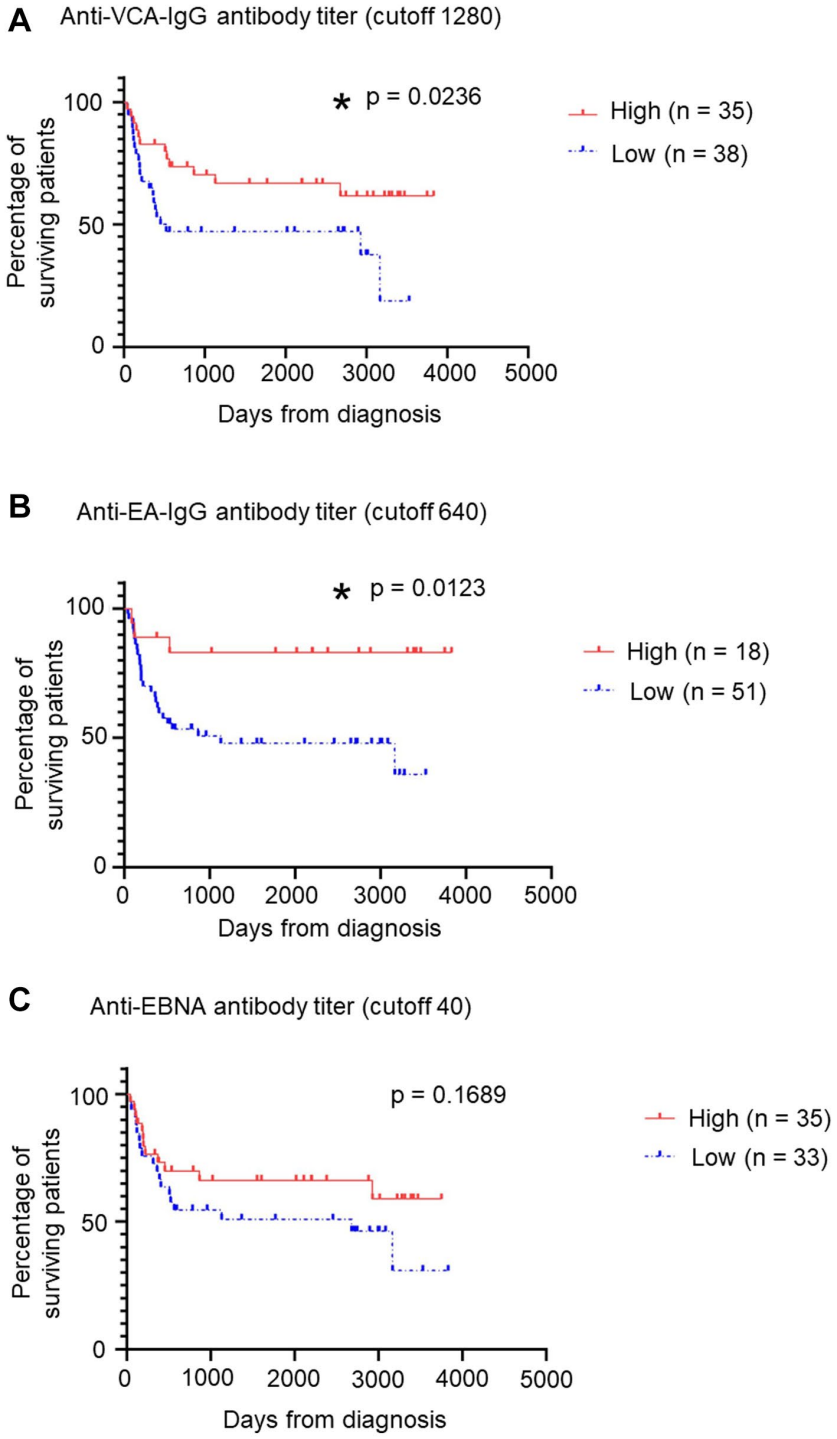
Relation between patients’ survival and some anti-EBV antibody titers. **(A)** Relation between patients’ survival and anti-VCA-IgG antibody titers using a survival curve. We established a cutoff value of 1,280. Red indicates the “High” group, including patients with >1,280 titers, while blue indicates the “Low” group. *p < 0.05 compared with the control. **(B)** Relation between patients’ survival and anti-EA-IgG antibody titers using a survival curve from GraphPad Prism 8. We established a cutoff value of 640. Red indicates the “High” group, including patients with >640 titers, while blue indicates the “Low” group. *p < 0.05 compared with the control. **(C)** Relation between patients’ survival and anti-EBNA antibody titers using a survival curve. We established a cutoff value of 40. Red indicates the “High” group, including patients with >40 titers, while blue indicates the “Low” group. EBNA, EBV nuclear antigen; EBV, Epstein–Barr virus; sCAEBV, systemic chronic active Epstein–Barr virus disease; VCA, viral capsid antigen.

Finally, we examined the relation between EBV antibody titers and prognosis in patients with sCAEBV. The distribution of anti-EBV antibody titers is shown in Supplementary Figure S5. Patients were categorized into two groups based on the titer of each antibody: the cutoff values were ≥ 1,280, ≥ 640, and ≥ 40 for the anti-VCA-IgG antibody, anti-EA-IgG antibody, and anti-EBNA antibody, respectively. The survival curves for the anti-EBV antibody titers are shown in Figure 3. The survival rates for the low-titer groups of the anti-VCA-IgG (Figure 3A) and anti-EA-IgG (Figure 3B) antibodies were significantly lower than those for the high-titer groups (*p* = 0.0236 and 0.0123, respectively). However, no significant difference was detected (*p* = 0.1689) between the low-titer and high-titer of the anti-EBNA antibody group (Figure 3C). Similarly, no significant differences were detected between the low-titer and high-titer of the anti-EBNA, anti-EA-IgG, and anti-EA-IgA antibodies groups when we set different cutoff values (Supplementary Figure S6). Significant differences might be obtained by increasing the number of samples.

## Discussion

To the best of our knowledge, this is the first report to analyze the significance of anti-EBV antibodies in patients with sCAEBV. We found that anti-EBV antibody titers were significantly different between patients and seropositive healthy controls. In addition, anti-VCA-IgG and anti-EA-IgG antibody titers were correlated with prognosis. These results suggest the hypothesis: anti-EBV antibodies are potent prognostic markers and may be the key to understanding the pathogenesis of sCAEBV.

Adult patients tended to have lower rates of positivity for anti-EA-IgA and anti-VCA-IgA antibodies (Figures 1B,E). IgA is an isotype antibody which is associated with local immunity within the mucosa. Anti-EA-IgA and anti-VCA-IgA antibodies are characteristically present in EBV-positive nasopharyngeal carcinoma (Liu et al., 2012, 2013; Tan et al., 2020). Thus, the presence of anti-EBV-IgA antibodies may reflect the presence of EBV-infected cells in the pharyngeal mucosa. Sometime after the initial EBV infection, adult patients may have fewer EBV-infected cells in the mucosa, resulting in a lower rate of anti-EBV-IgA antibody positivity.

In this study, we compared antibody titers between healthy controls, who were previously infected with EBV, and patients with sCAEBV. Serum EBV antibody titers and antibody positivity were compared between the control and age-matched AYA patients with sCAEBV, and significant differences in anti-EBNA and anti-VCA-IgG antibody titers were observed (Figures 2B–E). Patients with sCAEBV had lower anti-EBNA antibody positivity and lower anti-EBNA antibody titers than those in healthy controls. Anti-EBNA antibodies include all classes of antibodies: IgG, IgA and IgM. To investigate the clinical significance of anti-EBNA antibody titers in sCAEBV, future analyzes of each class of antibodies will be interesting. Interestingly, all anti-EBNA-negative sCAEBV patients were under 40 years of age. A higher occurrence in males and relatively low rate of anti-EBNA antibodies in childhood suggests the possibility of X-linked immune diseases. However, future analyzes are required to confirm these findings.

Another reason for the low anti-EBNA antibody positivity and low titers in patients with sCAEBV may be the low expression of EBNA in EBV-infected cells. EBV infection in T or NK cells is reported to be a type-2 latent infection without virus replication (Münz, 2019). EBNA1 is expressed in EBV-infected cells of type 2 latent infection. However, Iwata et al. (2010) analyzed *EBNA1* expression in 24 young patients with sCAEBV using real-time RT-PCR, and found that *EBNA1* expression was lower in the PB mononuclear cells (PBMCs) of sCAEBV patients compared to that in EBV-positive T or NK tumor cell lines. In addition, 14 of the 24 patients had undetectable *EBNA1* gene expression in the PBMCs (Iwata et al., 2010). In the present study, anti-EBNA antibody-negative cases were particularly common among younger patients who may have had low EBNA1 protein expression. Low EBNA1 protein expression in EBV-positive T and NK cells of patients with sCAEBV may contribute to host immune evasion mechanisms, resulting in the failure to eliminate EBV-infected cells and sCAEBV development. EBNA1 protein expression in EBV-infected cells and its regulatory mechanisms in sCAEBV should be investigated in a large number of patients.

Furthermore, EBNA-1 is an intracellular antigen. sCAEBV is associated with defects in cytolytic mediators (Fujieda et al., 1993; Shibayama et al., 2017), which lead to reduced apoptosis of EBV-infected cells. Intracellular antigens can be exposed on membrane blebs of apoptotic cells, where they can be recognized by the B-cell receptor of B cells and induce a B-cell and antibody response. Defective cytolysis could lead to lower EBNA1 exposure to extracellular milieu and lower antibodies formation and production.

While anti-EBNA antibody levels tended to be lower in patients with sCAEBV than in healthy controls, anti-VCA-IgG antibody titers were significantly higher in sCAEBV patients. VCA is a protein found in EBV viral particles (Johannsen et al., 2004; Wang et al., 2011); however, because the virus does not replicate in patients with sCAEBV, VCA is not detected in their PB (Münz, 2019). Therefore, we assumed that the high anti-VCA-IgG antibody titer did not reflect EBV levels. Polyclonal hypergammaglobulinemia, which is observed in patients with sCAEBV, is associated with persistent stimulation of B cells by activated EBV-infected T cells or NK cells (Wakiguchi et al., 2000; Okano et al., 2005; Nomura et al., 2011). In other words, anti-VCA-IgG antibody titers may reflect an enhanced nonspecific immune response activated by EBV-infected cells. Furthermore, higher anti-VCA-IgG antibody titers and anti-EA-IgG antibodies, which are also antibodies against EBV virus particles, were associated with significantly better prognosis in patients with sCAEBV. These results suggest that the patients’ comprehensive immunity influences the prognosis of sCAEBV. However, we could not determine the relationship between anti-EBNA antibody titers and prognosis. The positive rate of anti-EBNA antibodies varied widely by age, and the differences in sex preference and clinical findings by age indicate that the pathogenesis of sCAEBV differs by age. Thus, in future, the significance of the relationship between anti-EBNA1 antibody titer and prognosis should be analyzed in a large number of patients, according to age.

Anti-VCA-IgG and anti-EA-IgG antibodies are significantly higher in EBV-positive B-cell lymphomas, such as Burkitt lymphoma, Hodgkin lymphoma, and nasopharyngeal carcinoma, than in healthy controls (Xie et al., 2023). This study showed that sCAEBV patient specimens also had similarly high anti-VCA-IgG antibody titers. Interestingly, these EBV-positive B-cell lymphomas have higher anti-EBNA-IgG antibody titers than do healthy controls, whereas the anti-EBNA antibody titers of sCAEBV patients in this study were lower than those of the healthy controls. The mechanism for the lower anti-EBNA antibody titer and positivity in sCAEBV is not clear but is interesting compared to other EBV-positive B-cell lymphomas. The abovementioned hypothesis in sCAEBV needs further evidence to be proven.

This study had limitations. This was a retrospective analysis based on a questionnaire. The timing and clinical laboratories that measured the antibody titers were variable as described in the Materials and methods: they are validated and standardized but may have introduced a bias. In the future, the results should be verified by unifying methods and measuring simultaneously. In addition, the sample of healthy controls was limited to individuals in their 20s. Thus, whether differences in antibody positivity and titers between patients and healthy controls are also observed in other age groups, especially in adults, should be verified by inclusion of all age groups in the sample.

Recently, research on immunotherapies such as PD-1 antibody and T cell activation therapy is advancing, allowing EBV-positive T or NK-cell neoplasms to be targeted by treatment (Bollard et al., 2014; Kwong et al., 2017). Further studies, focusing on the immune system in a large number of patients, will be useful in understanding the pathogenesis and developing treatment methods for sCAEBV.

## Data availability statement

The raw data supporting the conclusions of this article will be made available by the authors, without undue reservation.

## Ethics statement

The studies involving humans were approved by the ethics committees of Tokyo Medical and Dental University, St. Marianna University School of Medicine, and Tokyo Kasei University. The studies were conducted in accordance with the local legislation and institutional requirements. The participants provided their written informed consent to participate in this study.

## Author contributions

MN: Conceptualization, Investigation, Project administration, Writing – original draft. MiS: Formal analysis, Investigation, Writing – original draft. MY: Investigation, Writing – review & editing. YK: Formal analysis, Writing – review & editing. AO: Investigation, Writing – review & editing. ES: Formal analysis, Writing – review & editing. IY: Resources, Writing – review & editing. MeS: Resources, Writing – review & editing. AA: Conceptualization, Funding acquisition, Project administration, Supervision, Writing – original draft.
