# Supplementary material for: Clinical significance of anti-Epstein–Barr virus antibodies in systemic chronic active Epstein–Barr virus disease
Source: Front Microbiol. 2024 Jan 8;14:1320292. doi: 10.3389/fmicb.2023.1320292 (PMC10800478; doi:10.3389/fmicb.2023.1320292)
Supplement: Supplementary file 1 [file Data_Sheet_1.PDF]

**Supplementary Materials**

**Figure S1. Flowchart of patient selection for this study.**

EBV, Epstein-Barr virus; sCAEBV, systemic chronic active Epstein-Barr virus disease;

**Figure S2. Relationship between sex and anti-EBV antibody titers.**

A. Samples of anti-EBNA antibody classified into six groups by age group and sex. Green indicates children, blue indicates AYA, and red indicates adults. The number of available samples is shown at the top of the graphs.

B. Samples of anti-EBNA antibody classified into six groups by age group and sex, and comparison of anti-EBNA antibody titers carried out using the Kruskal–Wallis test with post-hoc analysis.

C-D. Samples of anti-VCA-IgG and anti-EA-IgG antibody classified by sex, and comparison of each antibody titer carried out using the Kruskal–Wallis test with post-hoc analysis.

AYA, adolescent and young adult; EBNA, EBV nuclear antigen; VCA, viral capsid antigen; EA, anti-early antigen;

**Figure S3. Relationship between age and anti-EBV antibody titers.**

Samples of five anti-EBV antibodies classified into three groups by age group, and comparison of anti-EBV antibodies titers performed using the Kruskal–Wallis test with post-hoc analysis.

AYA, adolescent and young adult; EA, anti-early antigen; EBNA, EBV nuclear antigen; EBV, Epstein-Barr virus; VCA, viral capsid antigen

**Figure S4. Relationship between EBV-infected cell types and anti-EBV antibody titers.**

Samples of five anti-EBV antibodies classified into two groups by EBV-infected cell types (T-cell type and NK-cell type), and comparison of anti-EBV antibodies titers performed using the Kruskal–Wallis test with post-hoc analysis. T-cell type include CD4, CD8, γδT, and others (CD4 + CD8, CD8 + γδT). NK-cell type include CD56 and others (CD56 + CD56 (-)).

EA, anti-early antigen; EBNA, EBV nuclear antigen; EBV, Epstein-Barr virus; VCA, viral capsid antigen

**Figure S5. Distributions of some anti-EBV antibody titers were used to determine the cutoff values for survival analysis.**

Histograms showing the distributions of five anti-EBV antibody titers: anti-VCA-IgG, anti-VCA-IgA, anti-EBNA, anti-EA-IgG, and anti-EA-IgA.

EA, anti-early antigen; EBNA, EBV nuclear antigen; EBV, Epstein-Barr virus; VCA, viral capsid antigen

**Figure S6. Relations between the patients' survival and some anti-EBV antibody titers when we set other cutoff values.**

A–B. The relation between patient survival and anti-EBNA antibody titers using the survival curve derived using GraphPad Prism 8. We established a cutoff value of 10 to determine positivity or negativity and 160 to classify patients into two groups (high and low). The red line represents the positive/high group, while the blue line represents the negative/low group.

C–D. The relation between patient survival and anti-EA-IgG antibody titers using the survival curve derived using GraphPad Prism 8. We established cutoff values of 160 and 320. The red line indicates the high group, while the blue line indicates the low group.

E. The relation between patient survival and anti-EA-IgA antibody titers using survival curves derived using GraphPad Prism 8. We established a cutoff value of 10. The red and blue lines represent the positive and negative groups, respectively.

EA, anti-early antigen; EBNA, EBV nuclear antigen; EBV, Epstein-Barr virus; VCA, viral capsid antigen

## Figure S1

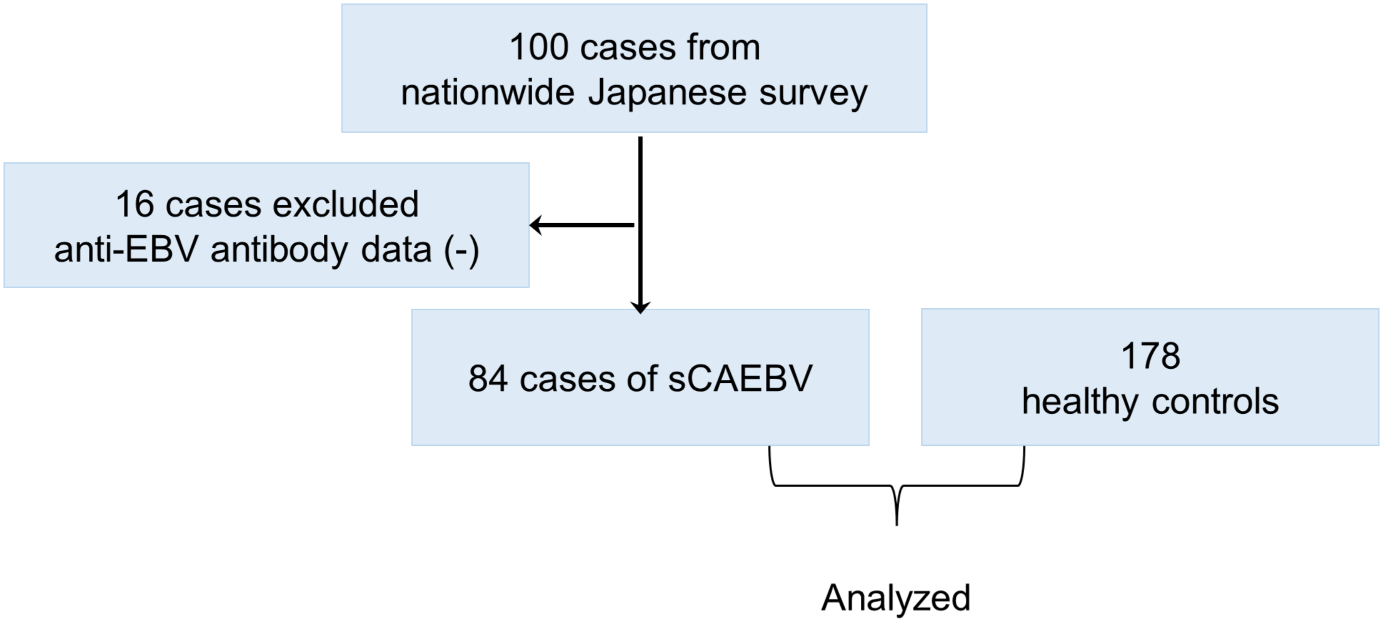

100 cases from nationwide Japanese survey
16 cases excluded anti-EBV antibody data (-)
84 cases of sCAEBV
178 healthy controls
Analyzed

# Figure S2

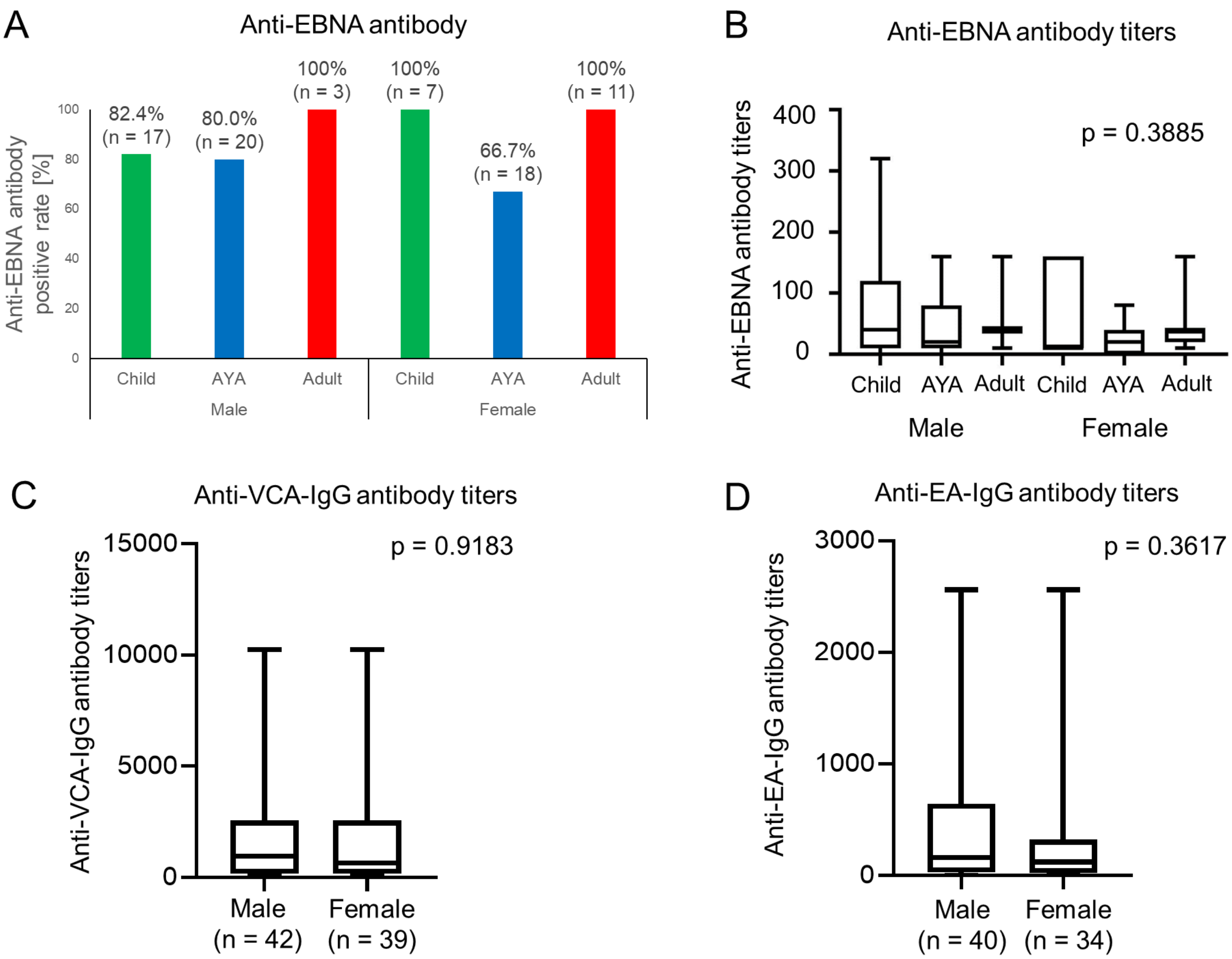
A
Anti-EBNA antibody
Anti-EBNA antibody positive rate [%]
100
80
60
40
20
0
82.4% (n = 17)
80.0% (n = 20)
100% (n = 3)
100% (n = 7)
66.7% (n = 18)
100% (n = 11)
Child
AYA
Adult
Child
AYA
Adult
Male
Female
B
Anti-EBNA antibody titers
Anti-EBNA antibody titers
400
300
200
100
0
p = 0.3885
Child
AYA
Adult
Child
AYA
Adult
Male
Female
C
Anti-VCA-IgG antibody titers
Anti-VCA-IgG antibody titers
15000
10000
5000
0
p = 0.9183
Male (n = 42)
Female (n = 39)
D
Anti-EA-IgG antibody titers
Anti-EA-IgG antibody titers
3000
2000
1000
0
p = 0.3617
Male (n = 40)
Female (n = 34)

# Figure S3

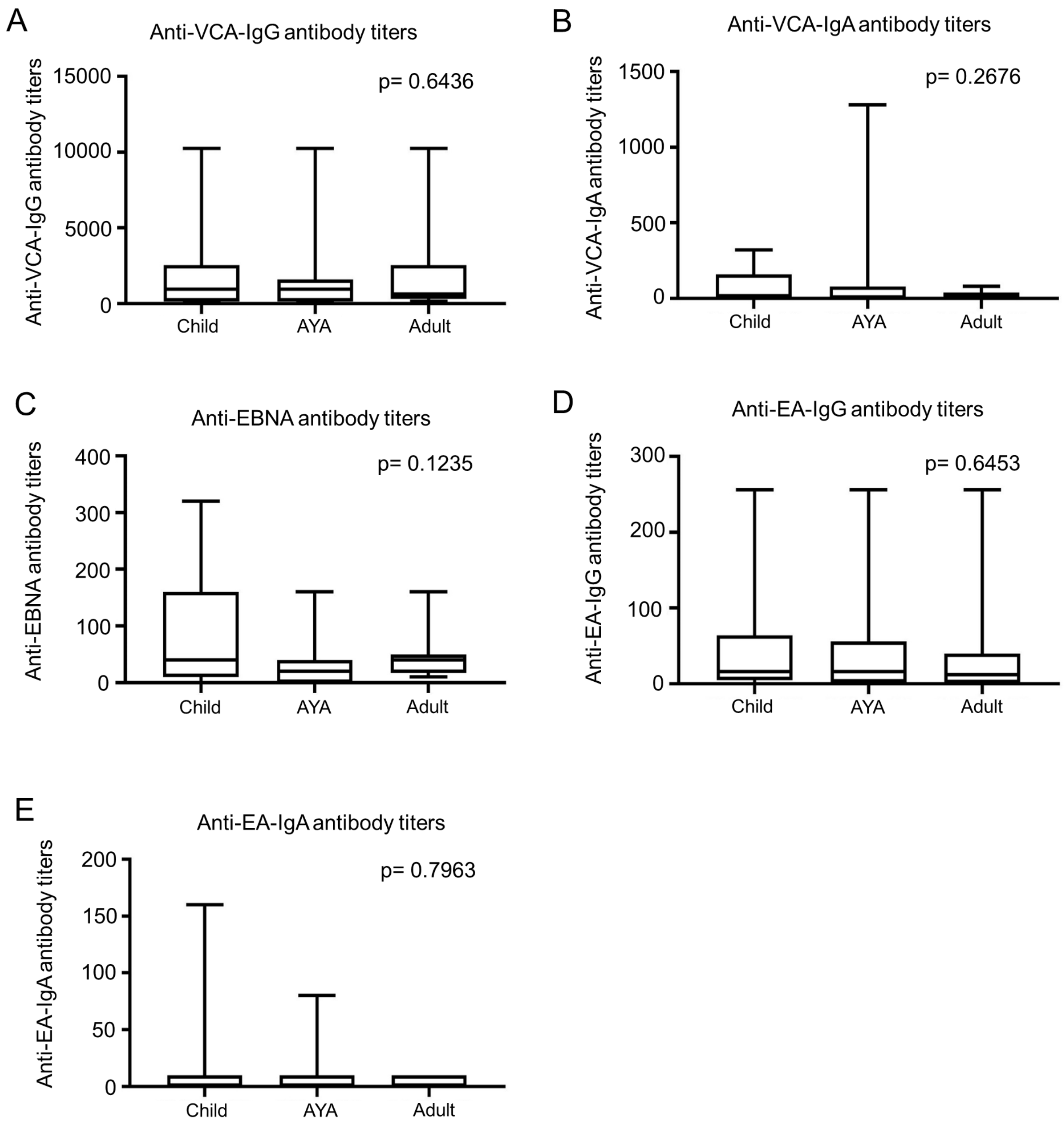
A
Anti-VCA-IgG antibody titers
p= 0.6436
Anti-VCA-IgG antibody titers
15000
10000
5000
0
Child
AYA
Adult
B
Anti-VCA-IgA antibody titers
p= 0.2676
Anti-VCA-IgA antibody titers
1500
1000
500
0
Child
AYA
Adult
C
Anti-EBNA antibody titers
p= 0.1235
Anti-EBNA antibody titers
400
300
200
100
0
Child
AYA
Adult
D
Anti-EA-IgG antibody titers
p= 0.6453
Anti-EA-IgG antibody titers
300
200
100
0
Child
AYA
Adult
E
Anti-EA-IgA antibody titers
p= 0.7963
Anti-EA-IgA antibody titers
200
150
100
50
0
Child
AYA
Adult

# Figure S4

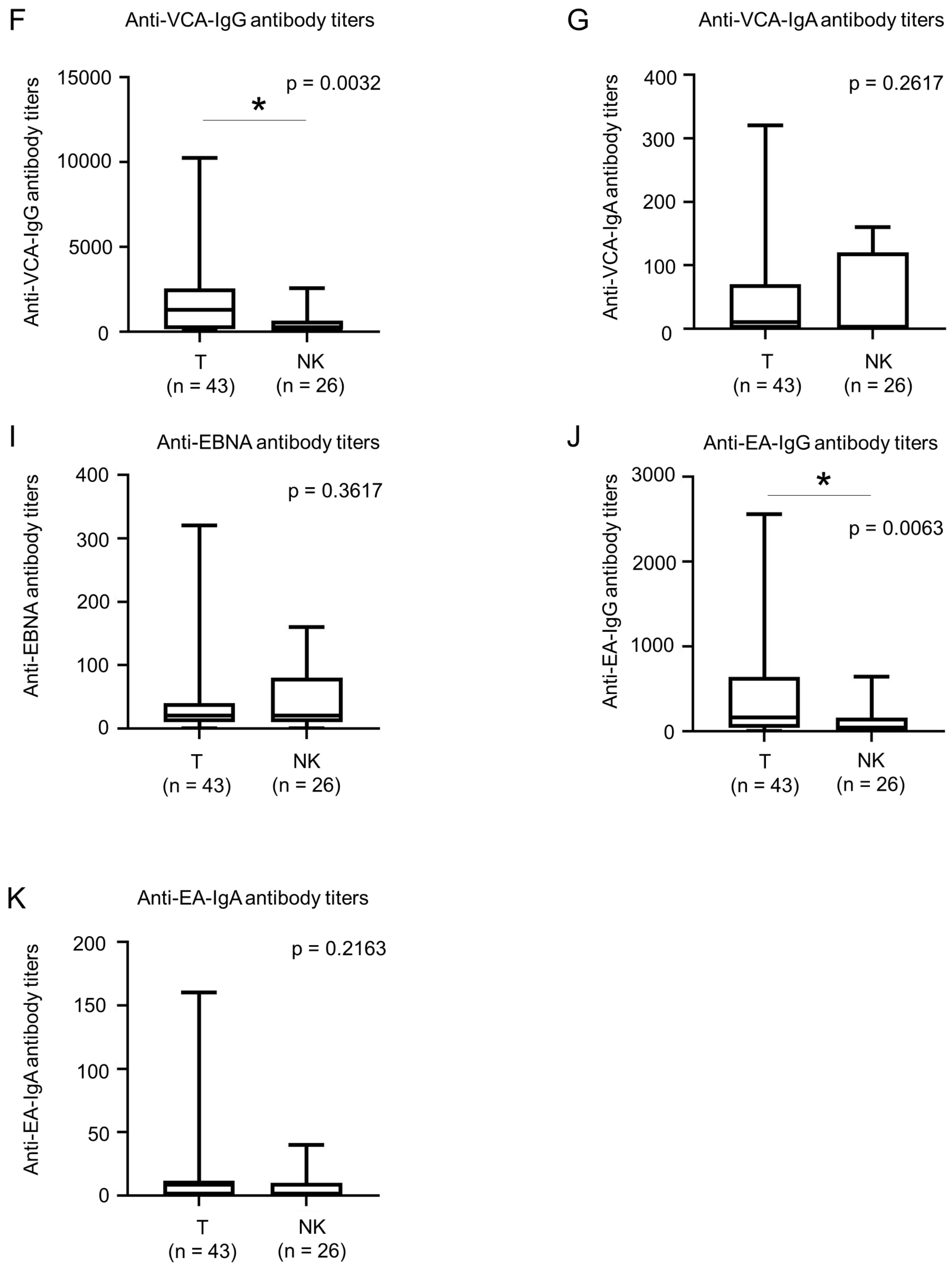
F
Anti-VCA-IgG antibody titers
Anti-VCA-IgG antibody titers
15000
10000
5000
0
p = 0.0032
*
T
(n = 43)
NK
(n = 26)
G
Anti-VCA-IgA antibody titers
Anti-VCA-IgA antibody titers
400
300
200
100
0
p = 0.2617
T
(n = 43)
NK
(n = 26)
I
Anti-EBNA antibody titers
Anti-EBNA antibody titers
400
300
200
100
0
p = 0.3617
T
(n = 43)
NK
(n = 26)
J
Anti-EA-IgG antibody titers
Anti-EA-IgG antibody titers
3000
2000
1000
0
*
p = 0.0063
T
(n = 43)
NK
(n = 26)
K
Anti-EA-IgA antibody titers
Anti-EA-IgA antibody titers
200
150
100
50
0
p = 0.2163
T
(n = 43)
NK
(n = 26)

# Figure S5

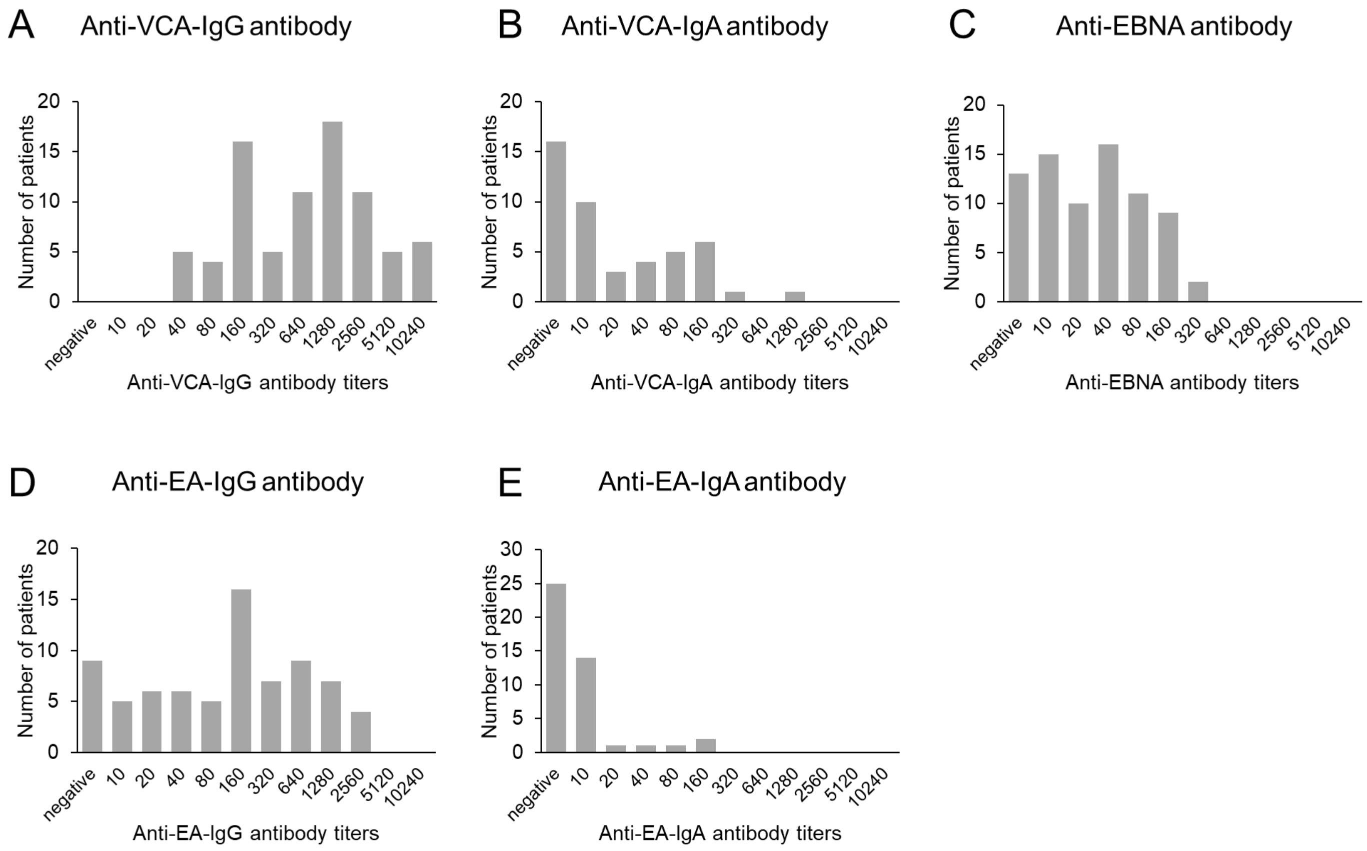
A
Anti-VCA-IgG antibody
Number of patients
Anti-VCA-IgG antibody titers
B
Anti-VCA-IgA antibody
Number of patients
Anti-VCA-IgA antibody titers
C
Anti-EBNA antibody
Number of patients
Anti-EBNA antibody titers
D
Anti-EA-IgG antibody
Number of patients
Anti-EA-IgG antibody titers
E
Anti-EA-IgA antibody
Number of patients
Anti-EA-IgA antibody titers
negative
10
20
40
80
160
320
640
1280
2560
5120
10240

# Figure S6

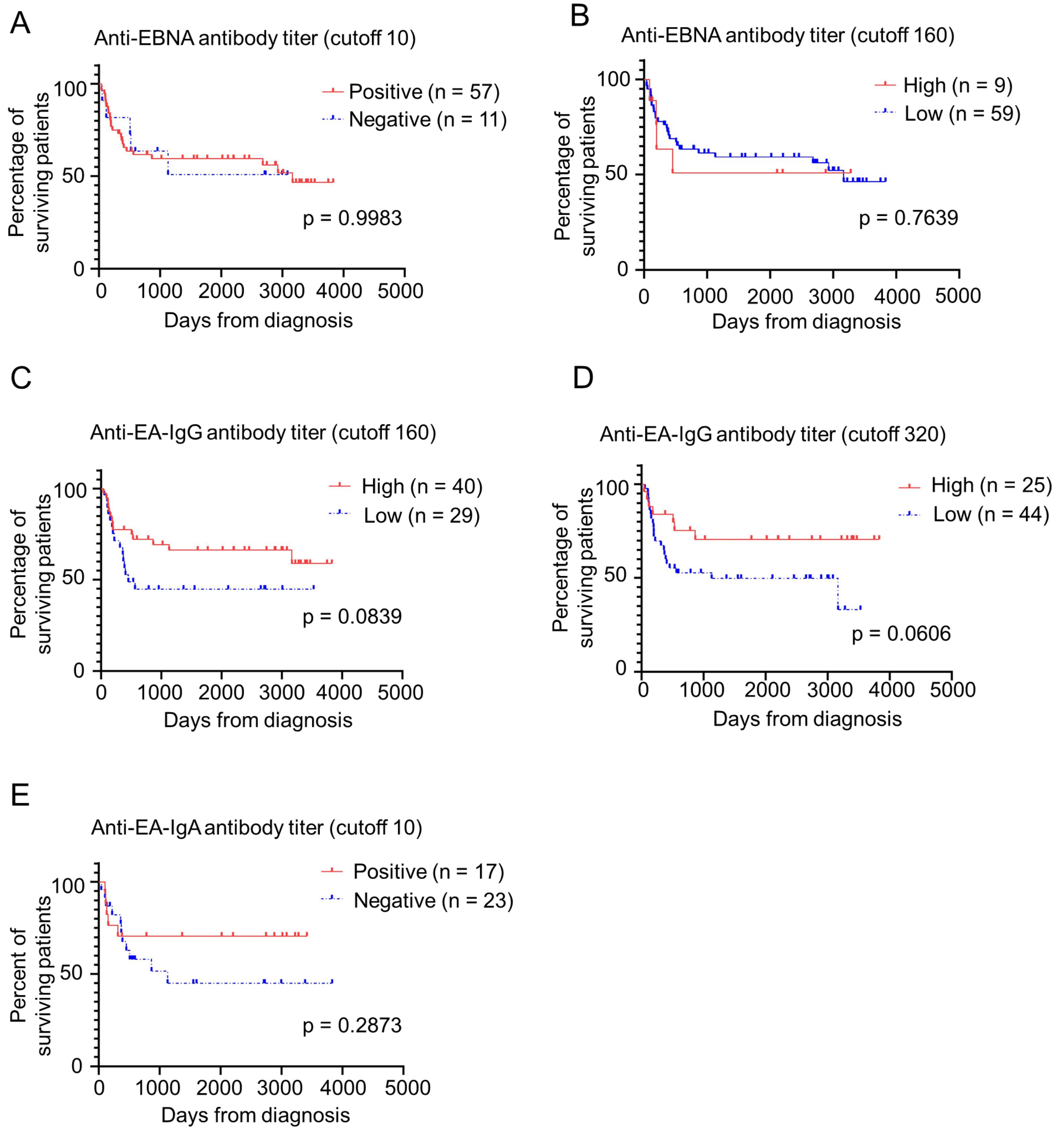
A
Anti-EBNA antibody titer (cutoff 10)
Positive (n = 57)
Negative (n = 11)
p = 0.9983
Percentage of surviving patients
Days from diagnosis
B
Anti-EBNA antibody titer (cutoff 160)
High (n = 9)
Low (n = 59)
p = 0.7639
Percentage of surviving patients
Days from diagnosis
C
Anti-EA-IgG antibody titer (cutoff 160)
High (n = 40)
Low (n = 29)
p = 0.0839
Percentage of surviving patients
Days from diagnosis
D
Anti-EA-IgG antibody titer (cutoff 320)
High (n = 25)
Low (n = 44)
p = 0.0606
Percentage of surviving patients
Days from diagnosis
E
Anti-EA-IgA antibody titer (cutoff 10)
Positive (n = 17)
Negative (n = 23)
p = 0.2873
Percent of surviving patients
Days from diagnosis
